# The old, the new, or the old made new? Everyday counter-narratives of the so-called fourth agricultural revolution

**DOI:** 10.1007/s10460-022-10374-7

**Published:** 2022-10-31

**Authors:** David Christian Rose, Anna Barkemeyer, Auvikki de Boon, Catherine Price, Dannielle Roche

**Affiliations:** 1 School of Water, Energy, and the Environment, Cranfield University, College Road, Cranfield, Bedford, MK43 0AL UK; 2 School of Agriculture, Policy and Development, University of Reading, Earley, Reading, RG6 6AR UK; 3 School of Geography, University of Nottingham, Sir Clive Granger Building, University Park, Nottingham, NG7 2RD UK

**Keywords:** Agriculture 4.0, Fourth agricultural revolution, Everyday, Micro-invention, Retro-innovation, Technology, Tinkering

## Abstract

Prevalent narratives of agricultural innovation predict that we are once again on the cusp of a global agricultural revolution. According to these narratives, this so-called fourth agricultural revolution, or agriculture 4.0, is set to transform current agricultural practices around the world at a quick pace, making use of new sophisticated precision technologies. Often used as a rhetorical device, this narrative has a material effect on the trajectories of an inherently political and normative agricultural transition; with funding, other policy instruments, and research attention focusing on the design and development of new precision technologies. A growing critical social science literature interrogates the promises of revolution. Engagement with new technology is likely to be uneven, with benefits potentially favouring the already powerful and the costs falling hardest on the least powerful. If grand narratives of change remain unchallenged, we risk pursuing innovation trajectories that are exclusionary, failing to achieve responsible innovation. This study utilises a range of methodologies to explore everyday encounters between farmers and technology, with the aim of inspiring further work to compile the microhistories that can help to challenge robust grand narratives of change. We explore how farmers are engaging with technology in practice and show how these interactions problematise a simple, linear notion of innovation adoption and use. In doing so, we reflect upon the contribution that the study of everyday encounters can make in setting more inclusionary, responsible pathways towards sustainable agriculture.

## Introduction

Prevalent narratives of technological change in agriculture predict that we are once again on the cusp of a new global agricultural revolution, driven by the need to grow more food, without damaging the environment (Barrett and Rose 2022; Duncan et al. 2021). The promises made by proponents of so-called agriculture 4.0, or the fourth agricultural revolution, tend to be ‘epochal’ (Miles 2019) and ‘deeply transformative’ (Duncan et al. 2021). Duncan et al. (2021, p. 1182) highlight several news articles promising that precision agriculture will have ‘one of the most pronounced impacts on ag production since the industrial revolution’, a prediction also noted in UK press coverage of agricultural technology (Barrett and Rose 2022). An emphasis tends to be placed on new, emergent technologies, such as Artificial Intelligence, robotics, gene editing, drones, and vertical farming (Klerkx and Rose 2020; Duncan et al. 2021). They promise to change production systems beyond recognition leading to increased productivity, reduced environmental damage, and socio-economic benefits. A socio-technical imaginary^1^ of linear, rapid progress is envisaged. Certain types of benefits are emphasised more than others, typically financial benefits of new technology, rather than issues such as farmer justice and environmental concerns (Duncan et al. 2021).

Discourses of techno-optimism in farming are nothing new, however, it is important to understand what the current dominant framings or socio-technical imaginaries (transformative, new, beneficial) of agriculture 4.0 set out to achieve. Birner et al. (2021) pose the question of who is driving the digital agricultural revolution? They explore stakeholders in the digitalisation of agriculture from large to small businesses down to user communities, finding that, although there are opportunities for small and medium sized enterprises, large, already powerful companies have more influence in setting the direction of travel. Duncan et al. (2021) argue that discourses of revolution frame transitions in a way that benefits those with existing power. Grand promises or socio-technical imaginaries of the future create hype and investment, as well as research and policy interest, in certain visions of the future (Jasanoff and Kim 2009; Borup et al. 2006). Hence, they can ‘guide activities’ towards achieving specific futures (Borup et al. 2006).

If the status quo is sustained in the food production system, this could reinforce existing inequalities; for example small-scale farmers, particularly in the developing world, are already less likely to be able to invest in new technologies due to expensive products, lack of accessibility to, or unaffordability, of data, and gender inequality (Mehrabi et al. 2021; McCampbell et al. 2021), as well as a lack of skills, as compared to larger businesses with the ability to support more diverse teams. Precision technologies, such as automated milking, have tended to lead to the decline of small farms and a re-structuring of the dairy industry towards a model of fewer, but larger farms (Vik et al. 2019). Likewise, some rural farm workers may not be able to retrain or retain value in an era of automation (Rotz et al. 2019), whereas smaller technology businesses may find it harder to scale innovations (Birner et al. 2021). New data-intensive technologies could reduce farmer autonomy and increase corporate control over farm decision-making (Brooks 2021), as well as widen inequities of land access (Duncan et al. 2022). In addition, ethical concerns over data ownership (Wiseman et al. 2019), animal welfare (Schillings et al. 2021), energy and material use (Streed et al. 2021) and health and safety (Basu et al*.* 2020) of emergent digital technologies have been highlighted.

Undoubtedly, therefore, a model of agriculture 4.0 that perpetuates capitalist, industrial agriculture will create winners and losers (Bronson 2019) and there is a burgeoning literature exploring this (see reviews by Klerkx et al. 2019; Fielke et al. 2020). Furthermore, Klerkx and Rose (2020) argue that a focus on game-changing technologies could divert attention away from the implementation of technologies and ideas that are ready now, instead favouring the development of emergent, untested products which may not realise their potential and may take several decades to be adopted at scale. The future of agriculture is not set and there are many potential visions of the future (Fleming et al. 2021; Ehlers et al. 2022). The choice of which pathways to pursue is an inherently political and normative choice (Klerkx and Rose 2020).

Duncan et al. (2021), Daum (2021) and Rose et al. (2021b) all note that counter-narratives to capitalist, intensive production could also be enabled by the use of the same, emergent technologies as well as low-tech and non-tech solutions. Agro-ecology, regenerative agriculture, ‘slow-tech’ (Duncan et al. 2021), farmer-made technologies (see e.g. the FarmHack movement^2^) and other alternative visions could be desirable to producer communities, as well as to wider society. Recent research on responsible innovation in agriculture argues that inclusion and reflexivity are vital to the setting of future trajectories; in other words, stakeholder views need to be taken into account and listened to when determining which types of agricultural future to support (Bronson 2019; Eastwood et al. 2019; Klerkx et al. 2019). If grand narratives of change, such as agriculture 4.0, are unchallenged and remain primarily focused on new, emergent technologies and modes of production not dissimilar from that of the past, we risk encouraging certain visions of the future over others without including farmers and other stakeholders in setting these visions. We risk, therefore, pursuing innovation trajectories that are exclusionary, failing to achieve responsible innovation.

Duncan et al. (2021) call for more research to bring forth counter-narratives of technological change in agriculture, adding to the growing critical literature on the digitalisation of agriculture (see reviews by Fielke et al. 2020; Klerkx et al. 2019). Further research by Baur and Iles (2022) calls on scholars to explore and critique the dominant technological frames, and the assumptions underpinning them, that have driven agricultural mechanization throughout history. This paper is inspired by these calls. Specifically, we explore how ‘everyday encounters’ (Glover et al. 2019) between farmers and technology challenge grand narratives of rapid change. We employ the concept of the ‘everyday’ as a theoretical anchor to interrogate the likely pace and directionality^3^ of technological change during agriculture 4.0 across different farms. Focusing on the everyday draws attention to how people engage with technology (Marres 2015). Whilst those who stand to profit from technology can potentially exaggerate the value of a new tool (Dauvergne 2020), everyday engagements reveal the potential pitfalls and overstated claims of grand narratives of change. For all, we show that a focus on the local, everyday enactment of technologies, as a complement to the global, pervasive narratives of technology revolutions, are fundamental to a more nuanced consideration of technological change.

## The everyday and histories of change

Before outlining our study, we introduce the theoretical grounding of this paper, as well as the research from rural sociology and history that we primarily draw on. Our purpose is to show how the concept of the everyday, alongside a conceptualisation of technological change in agriculture as one of evolution, rather than revolution, can help to elucidate the implicit and explicit effects of agriculture 4.0.

At the simplest level, ‘everyday’ technology refers to tangible objects that have become domesticated. Domestication research explores what happens to an artefact when a new technology enters the front door. Anthropological field research has been used as a way of exploring the minutiae of people’s engagement with new technology, such as family encounters with smart grid technology in Danish households (Nyborg 2015). This allows us to understand how people come to use technology and which technologies become domesticated so they are used every day and ultimately what the artefact means to people.

In a farming context, a characterisation used by Lundström and Lindblom (2021) helps us to consider what happens when technology enters the farm. The authors argue that researchers should consider how farmers ‘live with’ technology, rather than simply how they ‘act on’ it. The distinction is clear. Whilst studies of how farmers use technology have often been interested in the binary use or non-use of technology (*if* farmers use technology), they rarely concern themselves with *how* farmers use and interact with it. Yet, as Lundström and Lindblom (2021) show, observations of how different farmers use the same piece of technology over time will produce varying results. The process of *if, how,* and *why* technologies are used by a farmer, how these interactions change the farm environment, and how the farm environment changes these interactions, cannot be captured by binary notions of use or non-use. Rather, a two-way exchange occurs in which the artefact itself changes the social conditions into which it is launched, whilst users re-shape the artefact to suit their own lives. This re-shaping is influenced by the circumstances of individual farmers, for example their capacity to innovate, their situated knowledge (Lundström and Lindblom 2018) as well as their worldview (or imaginary) of why they farm and what they think farming is. As Gardezi et al. (2022, 15) write ‘[t]echnology always puts humans into a dance with it, and it is through these interactions that the agency continues to move like a pendulum between humans and machines’. An example of this is provided by Rose et al. (2018) who showed how computer-based decision support systems changed how farmers spent time in different places on the farm (e.g. more time in the office), but also how different individuals used the same piece of technology in varying ways; for example, some farmers found the system helpful and adapted management, whereas others ignored it if their experiential knowledge favoured a different approach. Each farmer had a different attitude towards technology use, partially informed by their worldview of what they thought farming was as a practice.

Documenting what Gardezi et al. (2022) term the ‘dance’ between farmers and technology requires a focus on the everyday and the creation of biographies. Using biographies of people and objects as a methodological approach in history is by no means new (though it is a novel approach within the context of agriculture 4.0 trajectories), as Gosden and Marshall (1999) have shown. By moving away from linear narratives and moving between multiple temporalities, we can observe historical processes from different and interesting angles across both agricultural and industrial systems. Despite the potential richness of researching the grounded, microhistories of technological change in farming, Kumar et al. (2017) argue that historians have failed to pay adequate attention to interrogating grand narratives of agricultural revolution. Indeed, they argue that historians have been rather silent on events such as the Green Revolution and may not have had much influence outside of the discipline. As we have articulated, however, there is clear value in documenting everyday experiences of technological change on the farm and in the construction of microhistories. Kumar et al. (2017) call for a more transdisciplinary approach to link sources of knowledge together and for collaborations between disciplines such as history, political science, sociology and economics. This call has been echoed elsewhere, for example, by Burchardt (2007, 465) who argues for a ‘new countryside history’ of change, ‘encompassing cultural and representational aspects’, rather than a ‘history of agriculture writ large’. Further, Baur and Iles (2022, 1) argue that we need to explore the history of agricultural mechanization and change; failure to do so, they argue, risks ‘retreading the same messy, conflict-laden, and unjust pathways of agrarian technological development.’

The documentation of microhistories of changes allows us to challenge deterministic myths of rapid technological progress. Historical narratives have frequently privileged design over use, and placed inventors above other agents who use, maintain, and repair technologies (Edgerton 1999). Popular academic narratives of agricultural change still focus on technology heroes, such as Norman Borlaug, and points in history where a dramatic revolution apparently occurred (e.g. the Green Revolution). While some research, for example by Lowenberg-deBoer and Erickson (2019), argue that adoption of some technologies like autosteer in tractors has been relatively quick, the overwhelming conclusion from the historical literature is that technological change in agriculture is characterised by steady evolution, and rarely, if ever, revolution (van der Veen 2010). Change is often marked by steady improvement, for example characterised by additions to existing technologies like adding better tyres to all-purpose tractors (McWilliams 1941), and cul-de-sacs or dead-ends of innovation (Kerridge 1969).

These myths endure because of the tendency for society to focus on visible moments of discovery, rather than the often long and complicated process of reaching the point of invention or ‘success’ (Shiva 2016). As Baur and Iles (2022) describe by tracing the development and implementation of a range of technologies in California between 1945 and 1980, including a tomato harvester, agricultural technologies often do not work at first and require a long process of change contributed to by farmers. They too argue that the long arduous process of development is often forgotten in later stories of success about the technology; and further, that we forget about the technologies that never worked and so forget that agri-tech development is non-linear and prone to failure.

In trying to understand how the myth of agricultural revolution pervades, Kerridge (1969), Pielke Jr. and Linnér (2019), Mcwilliams (1941), and others, take us to the idea of the ‘everyday’ without using the terminology. Kerridge (1969) and Pielke Jr and Linnér (2019) set out the purpose of revolutionary myths. In asking how the myth of revolution came to be borne with little evidence, Kerridge (1969, 475) states that ‘the strengths of belief depends in no way on evidence but on the …mind of the believer’. Critically analysing the myth of the Green Revolution, Pielke Jr and Linnér (2019) argued that the simplicity of the ‘technological sublime’ (Nye 1994) makes it beguiling. They write (page 19):

[f]amine averted by the intervention of scientific genius is a much more straightforward narrative than a famine-free story of incremental, accumulating, multi-factor progress in local agricultural production due to a complex tapestry of societal and political actors.

Baur and Iles (2022, 10) further this sentiment using an example of a tomato harvester, the implementation of which had negative consequences for some workers, a fact that is ‘strikingly absent’ from later accounts of its development:

The struggles of tomato workers to improve labor conditions, and later to simply keep their jobs, is strikingly absent from this history, we believe, not just because some scholars have been enamored by the glossy story of inventors and entrepreneurs who conjure a technological marvel against all odds, but also because the prime movers in this sociotechnical network were able to isolate their ‘technical’ work from the ‘political’ strife between workers and growers until the machine was already in the field.

Revolution is a straightforward narrative also because it hypes the value of technology ‘silver bullets’ to wicked problems that actually require much deeper socio-economic and political change and may create winners and losers in the process (Rose et al. 2021a). But, in the words of Baur and Iles (2022, 10) innovation ‘rarely progressed as smoothly, inevitably, or naturally as it sometimes appears in retrospect’.

Rather, non-linear agricultural innovation can proceed in different ways. For example, technologies could be implemented on-farm, but quickly disadopted, before becoming useful again at a later date (de Oca Munguia et al. 2021). Technologies could break, fall into disrepair, be disregarded (Schaffer 2011) or they could be given a new life through the process of retro-innovation (Zagata et al. 2020) or micro-invention (van der Veen 2010). Baur and Iles (2022) use a Social Construction of Technology approach to investigate how agricultural technologies both shape, and are shaped by, farmers and farm environments. There is growing interest in the process of retro-innovation on farm and the agency of users to re-shape technology, with farmers re-purposing older technologies for a new purpose, strapping different artefacts together and making something new (Zagata et al. 2020). The Farm Hack website,^4^ for example, illustrates a range of technologies made by a worldwide community of farmers who ‘build and modify’ their own tools. The community embraces ‘the long-standing farm traditions of tinkering, inventing, fabricating, tweaking, and improving things that break’. The website contains a forum for farm innovators to exchange ideas. Examples of technologies include a home built no-till seed drill,^5^ a water wheel transplanter,^6^ and a ‘make-your-own’ set of weigh scales for livestock.^7^

Old technologies can, therefore, be just as powerful as something new (Edgerton 2006; Vinsel and Russell 2020). The process of ‘tinkering’ (Nutch 1996) can mean that a piece of technology can end up bearing little resemblance to its original design (de Laet and Mol 2000), but understanding this journey requires a close engagement with its everyday use and re-use. Such a process of non-linear change has been seen as a counter to grand narratives of modernity and industrialisation in agriculture (Zagata et al. 2020), which widely speak about the importance of ‘new’ tools (Duncan et al. 2021).

We build on the work of Zagata et al. (2020) and Duncan et al. (2021) through a mixed methods study of everyday encounters between farmers and technology. Drawing on the aforementioned research in rural sociology and rural history, we explore how farmers are engaging with technology in practice, how they ‘live with’ technology (Lundström and Lindblom 2021), and show how these interactions problematise a simple, linear notion of innovation adoption and use. In doing so, we reflect upon the contribution that the study of everyday encounters can make in setting more inclusionary, responsible pathways towards sustainable agriculture.

## Methodology

Our overall objective was to explore everyday encounters between farmers and technology through a UK case study, a country in which agriculture 4.0 is being actively promoted by industry and policy-makers (Barrett and Rose 2022 and see the 4AR podcast^8^). We set out to document grounded experiences of innovation, as well as the pace and directionality of change. Several research questions guided the methodology for this paper:

1. Is agricultural technology necessarily seen by practitioner communities as something new?
2. What makes agricultural technology useful in practice?
3. How do practitioner communities see the future of agricultural technology?

The answers to these questions allow us to compile a narrative of change, which may or may not counter the dominant narrative of agriculture 4.0.

**Fig. 1:**
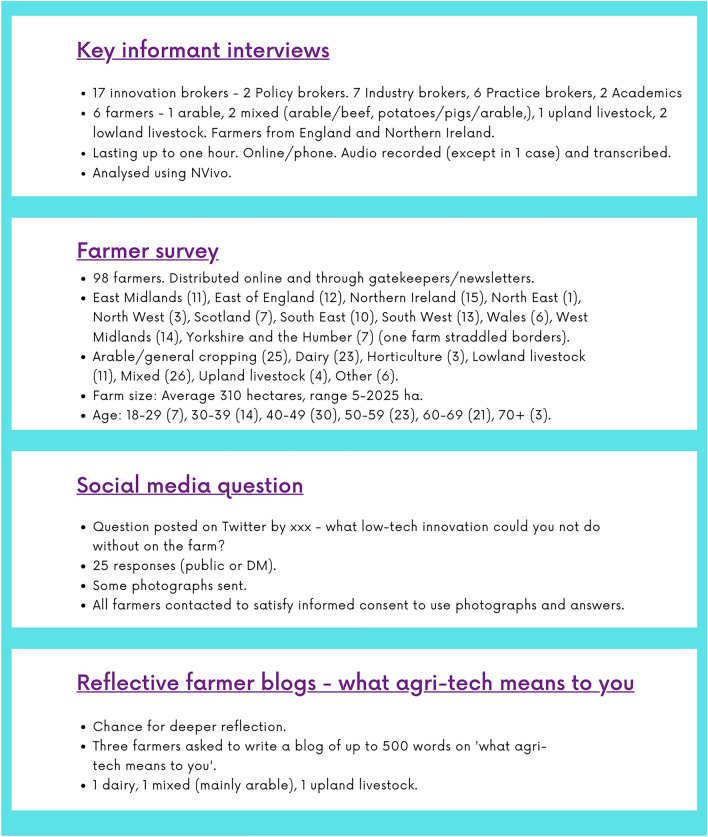
Mixed methods approach

Based on practicalities during COVID-19, we adopted a mixed methods approach and included both farmers and innovation brokers in our study (see Fig. 1). Whilst these innovation brokers, who performed a variety of policy, industry, and practitioner-facing roles in the UK’s Agricultural Knowledge Innovation System are somewhat divorced from the everyday farmer experience of technology, the literature has shown that innovation brokers can play a key role in technological change on farm (Ingram 2008; Cofré-Bravo et al. 2019; Rijswijk et al. 2019). They can play a key role in setting the direction and pace of technological change by influencing funding, policy, and practice, such that they have a role to play in determining what the everyday looks like on farm. We conducted a purposeful sample of different types of innovation brokers – those who worked with policy-makers and funders, those who worked in farming organisations or in the technology industry, and those who worked more closely with practitioners, for example by advising them about adoption of innovation. All research questions were covered by each method. All methods were approved by the University of Reading Ethical Committee.

We used an online survey of farmers to generate a large number of responses. We posed three open-ended questions to ensure that answers were not constrained. We asked farmers to define agricultural technology, to list up to five pieces of technology that were most useful on their farm and why, and to envision how their use of technology may or may not change on the farm by 2030. These questions were analysed as a means of exploring the three research questions above. This survey was distributed online and by gatekeepers via email, newsletters and farming forums, attracting 98 responses from a range of farm types and regions in the UK (see Fig. 1 for a breakdown). The survey responses are not intended to offer a representative view of UK farmers, but nevertheless offer a snapshot into the different everyday engagements between them and technology. We also asked farmers to leave contact details if they wished to take part in a short follow-up interview.

We interviewed farmers and innovation brokers in a semi-structured way. In total, we contacted twelve farmers based on purposive sampling and conducted six in-depth farmer interviews. These farmers, located in Northern Ireland and England, covered a range of arable and livestock enterprises. Interviews were conducted on the phone or online, lasting up to one hour, and transcribed fully where possible (not possible in one case due to a bad line, notes taken). The purpose of these interviews was to explore in-depth everyday farmer experiences of technology. We asked them to reflect on what agri-tech meant to them, the types of technologies that they found most useful on their farm, their visions of the future, as well as factors that affect technological change such as barriers to adoption and reasons for updating technology. We also explored concepts of retro-innovation and farmers’ perception of high- versus low-tech. Photographs of farmer inventions were supplied by some farmers and are utilised anonymously in this article with permission. As was also done as part of the social media question, farmers were asked to submit photographs as this can enable participants to express themselves in a different way, which may allow the researcher to get closer to everyday life (Oldrup and Carstennsen 2021). These interviews were fully transcribed.

We also chose to interview seventeen innovation brokers, the breakdown of which is in Fig. 1. We asked them a reduced amount of questions for this study due to their partial disconnection from the everyday (other questions asked were not part of this study), but still asked comparative questions to the farmer interviews. We focused on what agri-tech meant to them, the sorts of technologies they thought were most useful on-farm, and their visions of the future. These interviews were conducted online or on the phone and transcribed, lasting up to an hour.

To gain further depth on farmers’ everyday experiences of agricultural technology, three farmers wrote reflective blogs on what agri-tech meant to them. These farmers were selected from those who left details on the online survey (farmer blog writers were not also interviewed). The only instruction given to these three farmers was that the blog should be no longer than 500 words. The farmers (dairy, mixed [mainly arable], upland livestock) agreed to publicly release these blogs to help recruit further participants to the survey once it had been launched.

Finally, we asked a question on Twitter via the handle of the lead author. To explore the types of ‘lower-tech’ technologies that farmers found useful every day on the farm, we asked farmers to identify examples of useful low-tech innovations, either in writing or via a picture. 25 responses were given publicly or via direct message and respondents were asked if pictures could be used in the final publication.

All qualitative data were coded thematically through a process of manual coding. Whilst there is no universal method of coding interviews, the method outlined by Bryman (2008) provides an account of how it may be practised, which we followed: (1) Open coding—the initial classification and labelling of themes based on words in the transcript, (2) Axial coding—a reanalysis of open coding aimed at grouping like themes together and then (3) Selective coding—an identification of the most important themes in the study.

We acknowledge some limitations of the study. Firstly, we made use of online methods in recruiting farmers to the survey and identifying farmers to interview and to write reflective blogs. We know that there is a digital divide in rural areas in terms of skills and infrastructure (Hurley et al. 2022) which prevents some farmers from making optimal use of certain types of digital technologies. Conducting the surveys and interviews online may mean that we missed the views of less digitally-connected farmers, although evidence from the survey and interviews suggests that we did not just talk to farmers using ‘higher-tech’ digital technologies. Secondly, we acknowledge the relatively low sample size of farmers from interviews and the survey. The purpose was never to collect a representative sample of UK farmers, but rather to explore a range of possible experiences of technological change. To this end, we achieved responses from a range of regions, farm sizes, ages, and farm types, in the UK, although there were caveats (e.g. the age profile appears to be at the younger end of the spectrum for the UK average). Likewise, with the interviews of innovation brokers, we acknowledge that the purposeful sample may have been selective, although we managed to include a range of different types of broker (policy, industry, practice).

We acknowledge that ethnographic approaches tend to be best at observing how individuals interact with technology in their daily lives. Research on the domestication of technology, such as the study by Nyborg (2015), tends to use ethnographic methods, but they have also been used to explore digitalisation of agriculture (Prause 2021) and non-linear adoption pathways on farm (Smith et al. 2021). We consider that an ethnographic approach, spending time with farmers to observe how they interact with farm technologies, would be a valuable method to explore everyday technological change on-farm. However, in the context of the COVID-19 pandemic and associated social distancing, it was not deemed practical to spend time on farm in close proximity to farmers.

## Everday counter-narratives of agriculture 4.0

With the three aforementioned research questions in mind – exploring (1) whether agricultural technology is necessarily viewed as something new, (2) what makes agricultural technology useful, and (3) what the future of agri-tech looks like? – we discuss results in three corresponding sub-sections.

### New, old, or the old made new?

Dominant narratives of agriculture 4.0 predict the use of emergent game-changing technologies, such as Artificial Intelligence, drones and robotics on-farm. We asked innovation brokers and farmers to reflect on what agri-tech meant to them and whether useful technologies were new, old, or a mixture of the two.

**Fig. 2:**
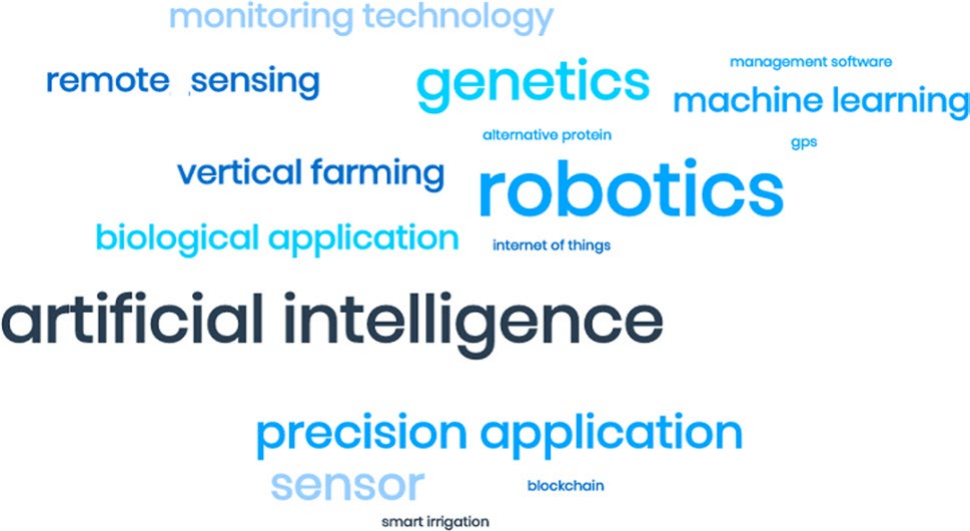
Word cloud based on innovation broker interview responses asking to identify the most important pieces of agricultural technology. Larger words mean mentioned more often

Though innovation brokers are partially divorced from the everyday, their interview responses tended to associate ‘agri-tech’ with more emergent technologies. Figure 2 shows a word cloud emphasising the technologies mentioned most often when innovation brokers were asked which types of agri-tech were most useful.

An innovation broker who regularly liaises with farmers about innovation on their farms, however, noted some caution with placing an undue emphasis on emergent technologies as the most useful. They argued:

Everyone thinks [agri-tech] needs to be wacky and new but it doesn’t at all. It doesn’t have to be new and wacky…what’s innovative to a poultry unit will be very different to what’s innovative to a small ruminant farmer, or sheep hill farmer. So, innovation is very different to different people. (Broker 10)

**Fig. 3:**
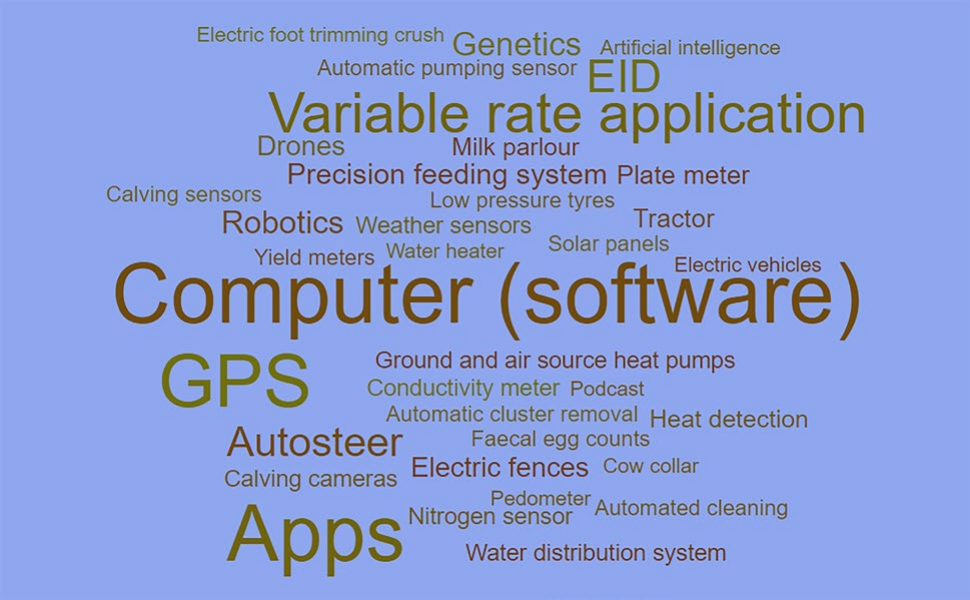
Word cloud based on farmer responses asking to identify the most important pieces of agricultural technology on their farm. Larger words mean mentioned more often

Answers from the farmer survey gave a larger range of the most useful technologies on the farm as shown in Fig. 3. Examples included emergent technologies, such as Artificial Intelligence and robotics, but mainly covered a range of different technologies that have been available for much longer – ranging from a basic tractor, electric fence, a computer, autosteer, to smartphone apps. It is clear from Fig. 3 and interview discussions that useful technology is not always something that is new.

**Fig. 4:**
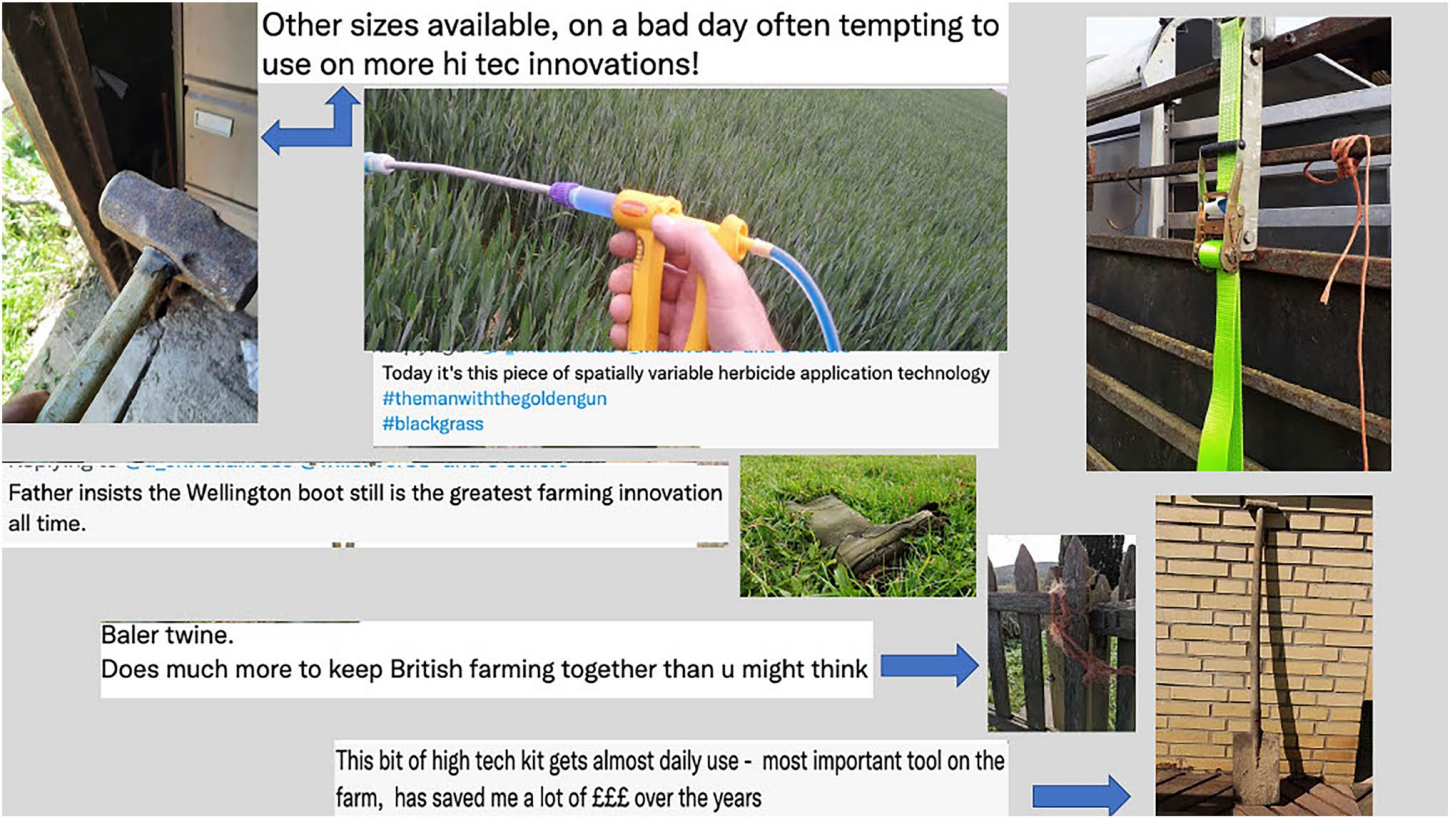
Examples of vital low-tech innovations given by farmers on Twitter

In response to the social media question asking which low-tech innovations were most useful on the farm, there were several visual and written comments shown in Fig. 4. All demonstrate the enduring everyday value of several pieces of older technology, certainly as compared to many of the technologies being tacitly associated with agriculture 4.0. We return to the pervading usefulness of older technologies in the following section.

In addition to the old versus the new, inspired by historical work on the non-linearity of technological change in farming and historical and more recent work on tinkering (Nutch 1996), micro-invention (van der Veen 2010), and retro-innovation (Zagata et al. 2020), interviews asked farmers to reflect on the process of technological change on the farm. We explored questions such as why and when they decided to repair or replace ‘old’ technologies and whether they adapted existing technologies for the same or a new purpose.

The value of older technologies, noted in previous literature by Vinsel and Russell (2020) and Edgerton (2006), was stressed repeatedly in the farmer interviews, mainly because much of it still worked and performed the intended task well. Farmers were keen to stress that “*we can learn from the past*” (F3). One farmer noted that they were still using the tractor bought in 1994 and it goes out every single day. Tractor registration dates given in three interviews were 1994, 2005, and 2010. Another farmer was able to talk about the daily use of a technology from 1954:

So my favourite tool is an elevator. Now that piece of machinery is from 1954, I think. So it's like getting on for 70 years old. (F3)

Another agreed saying that they “couldn’t really survive without the tractor, as it works every time” (F4), even the ones without the new gadgets on them. One farmer said:

…a plough. You may regard this as old technology because what is in at the moment is a direct drill. We found that the machines that did this, the soil structures didn’t cope terribly well. We also began to have weed issues as a consequence of doing it and so we reverted back to the plough. So, a plough lot of farmers would think of as an old technology as has been around for 2000 years, but I rather like it. It has its place (F2).

Change in farming has sometimes occurred through a re-invention of the old into something new or indeed simply the re-discovery of old principles adopted in the present without change. Looking backwards has sometimes provided the solution to problems that may not have been found simply with a cursory glance to the future. In our study, farmers gave numerous examples of where they had made their own or adapted existing technologies for a specific purpose. One farmer, on the one hand praising the innovativeness of micro-invention, whilst also downplaying its significance, said that their partner was skilled at making things for themselves:

My husband is very mechanically minded and he will modify stuff… He made me irrigation system for my vegetable garden…we bought the stuff on the Internet and we now have an irrigation system…. Or we had a gate that we open every morning to go down the farm. But it would always swing back shut. So we made like a little metal clasp that you can shut the gate with. (F7)

They continued:

necessity is the mother of invention…as you do things and things don't work, he'll think of a way around it…You know, the plants were dying in the vegetable garden, and I couldn't get the hose around there…I didn't want to stand there for hours with watering cans. […] […] So he thought, OK, we'll try and find an irrigation system. So he gets around it. Usually a problem, then think about it, then come up with a solution. Or maybe you see someone else doing it, or you copy an idea. Something you've seen someone else do.

A further farmer looked towards the coming months as an opportunity to fix a chisel plough that was not working effectively:

My chisel plough didn’t actually work so that was a big problem, the idea was great and the machine was great but the shear pins that were controlling it just would not stand the strain of the work, which was frustrating, so that’s a little project for this winter but I’m not as good at that sort of thing as many of my contemporaries. (F2)

An additional two examples were given in a different interview.

We’ve built variable tillage tools in the past, different types of irrigation pumps that can feed at different volume rates, so that we can save money on irrigation pumps. We added inverters to our store fans in early 2000s to be able to modify airflow. (F3)

**Fig. 5:**
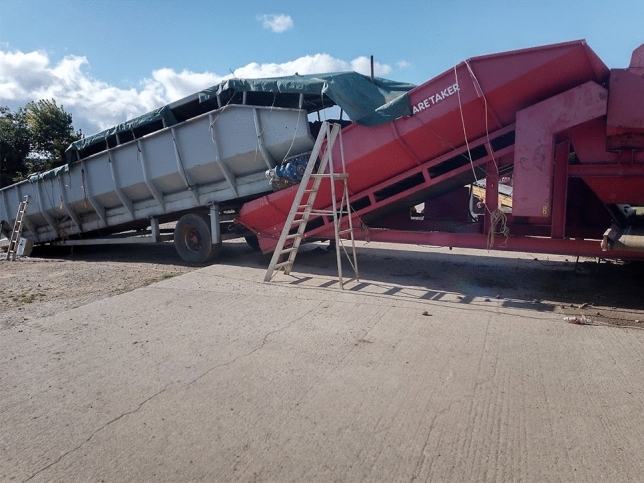
Farmer invention: feed potato grader to hold potatoes. Farmer description:“ The grey hopper holds 14tons of potatoes and was previously part of a carrot line in a supermarket packhouse. We built the chassis and fitted the axle that its now on to make it mobile and suitable to fit into our system. It starts and stops automatically via an ultrasonic sensor to feed the grader on demand, and its capacity has saved us a tractor, trailer and operator all season, worth today about £400/day. It cost £11,500 to build including the modifications

**Fig. 6:**
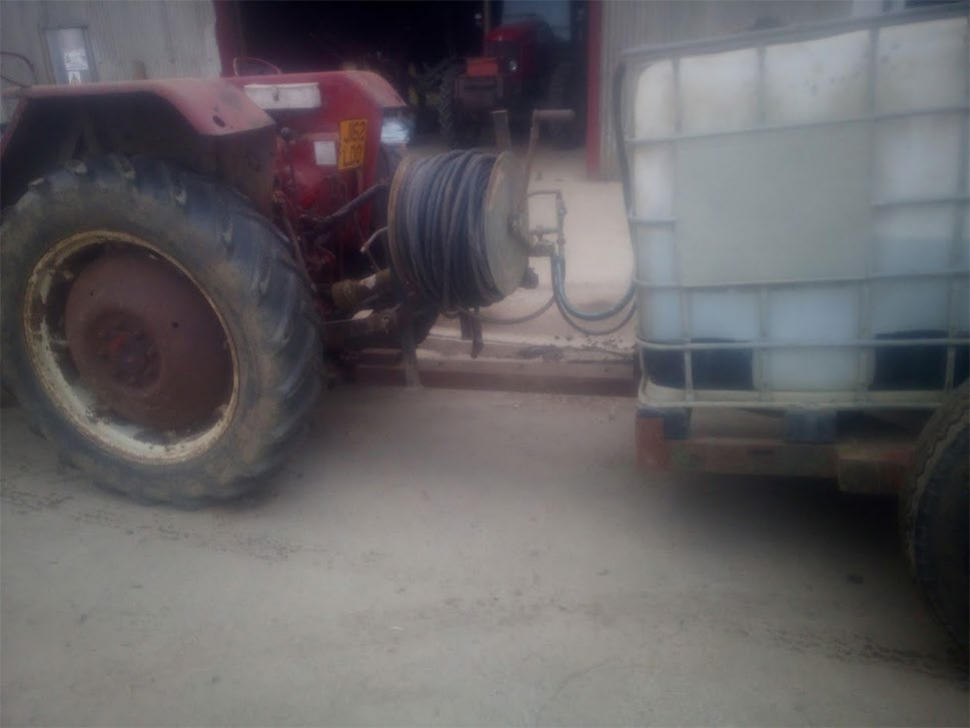
Farmer invention: Water cart for drain jetter. Farmer description: “We used a scrap beet cleaner bridge axle to make a trolley to carry two 1000L ibc tanks to hold water to feed the drain jetter/power washer fitted on the tractor linkage. It didn't cost much and saves a lot of chasing about and hassle. Easy jobs get done, hassle stops them being done!”

**Fig. 7:**
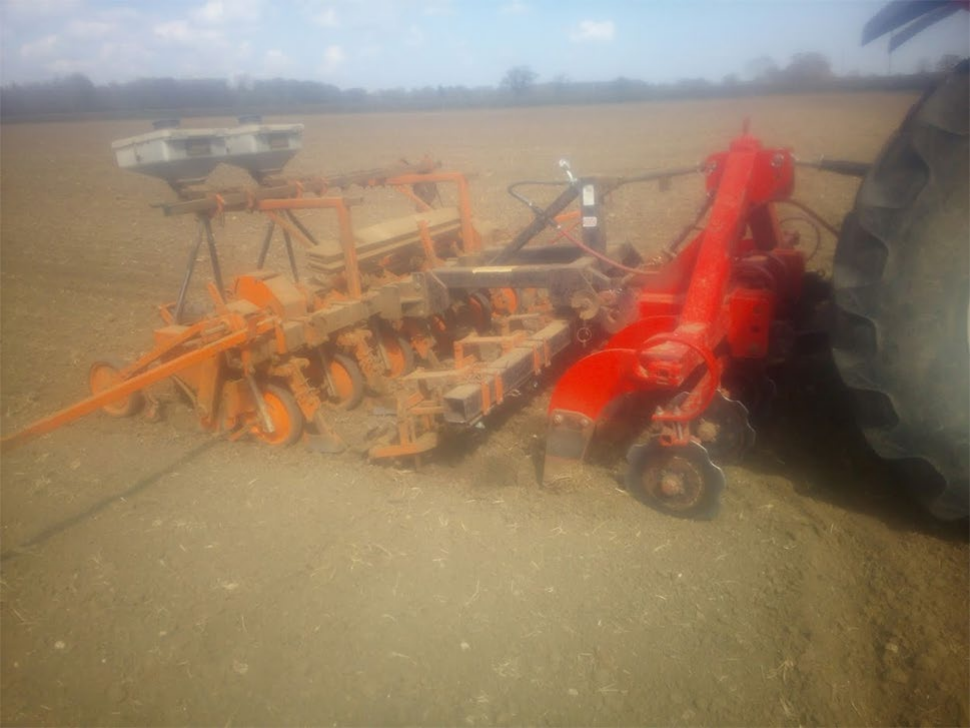
Farmer invention: beet drill time bar. Farmer description: “The red bar started life in front of a potato planter, then was recycled to carry six compaction removing legs, with leading discs sourced from a drill breakers yard. The middle section (row crumbler) came from an auction, under which is a spray bar from a redundant sprayer, applying a biostimulant to promote better rooting. The tank on the front of the tractor was previously used 12 years ago with a sprayer we had at the time. The micro granule applicator on the back of the drill was used by a previous owner for nematicide and is now used to sow crimson clover seed between the beet rows to attract beneficial insects that eat the virus carrying aphids that infect sugar beet.”

**Fig. 8:**
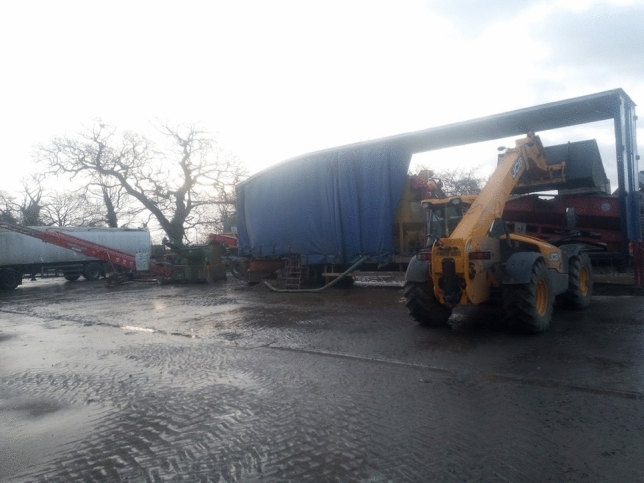
Farmer invention: Mobile potato washer. Farmer description: “This is the biggest of our recent projects, a mobile potato washer. Most washers need a hopper and elevator to feed them, and so are static. We built one into a 45' curtain-sided step frame trailer. A second-hand donor grader provided a sensor-controlled hopper and coils and all of the cross conveyors. This is fed directly with the forklift, the coils remove any loose soil, then they go up a flighted elevator surplus from a redundant box filler on farm, into a 3 × 1 m barrel washer sourced from a redundant packhouse in Ireland. On exit they are rinsed onto a picking table and head out through the headboard of the trailer into another flighted elevator which feeds them into a remote-controlled telescopic elevator and ito the lorry. The green section in the line is a destoner, which we put in the line for various customers, but not all. The pickers are protected from wind, rain and cold, and now have much more comfortable working conditions…Cost £42,000. Saving minimum £25,000/year.”

Another farmer gave several examples of things they had made, largely by re-purposing second-hand equipment (Figs. 5, 6, 7, 8). Each of their creations are described and pictured in turn below (with the permission of the farmer):

There are thus plenty of everyday encounters between farmers and technology that involve the old becoming the new; an older piece of technology adopting a new purpose due to modification, in the same way as the tools exhibited on the Farm Hack website. Other farmers in interview, however, noted that they were not skilled in adapting or making their own technologies, which illustrates that retro-innovation is not occurring on all farms. Noting the interesting use of the word ‘bodging’, one farmer said:

I am not very good at that type of thing. I’m not mechanically or technologically minded so nothing springs to mind and if it was it wouldn’t come into technology terms it would come into “bodging” terms. Nothing I would call something that some of these farm guys make, they’re really good at just making things, but I’m not. (F1)

### The shock of the old and the delusion of the new

Research both inside and outside of agricultural settings has illustrated the often surprising or ‘shocking’ (Edgerton 2006) value of older technologies (Lee and Vinsel, date; Zagata et al. 2020) and also addresses the challenges of implementing new technologies (Rose et al. 2016; da Silveria et al. 2021; Vik et al. 2021). There are, therefore, a myriad of potential reasons why agricultural transitions will not just be characterised by rapid, linear implementation of emergent technologies. An exploration with everyday encounters between farmers and technology helps to explain the enduring value of the old, as well as why newer technologies can take a long time to scale.

Firstly, our interviews, farmer blogs, and survey respondents shed light on why older technologies were still considered valuable, even if it appeared that something new would supersede them. Figure 4, for example, shows farmer quotes praising Wellington Boots as the ‘greatest farming innovation of all time’ or a spade as ‘the most useful tool on the farm’. Reasons for the enduring value of older technologies included:

#### Performance

Older technologies persisted if they performed well. Referring to a farming television series on Amazon Prime, an upland farmer wrote “Jeremy Clarkson discovered a drone will not replace a good sheepdog” (Blog 3) because it did not work as well. Another farmer in the survey spoke disdainfully of new technologies as “expensive things that haven’t replaced a notebook”. Thus, a piece of technology is still useful if it can stand the test of time and may not even be improved by newer modifications. One farmer said:

The basic pieces of kit that have stood the test of time, the combine harvester. When I look at my newest kit, the direct drill, the key elements of putting the seed in the ground is still the same but they’ve just put a GPS on top of it now and I do question how much more effective that really is. I’m not convinced it is any better than the wheel before, actually I think it is worse because it is slower to react than the wheel and the chain with the mechanical drives (F1).

One of the farmer reflective blogs, however, discussed whether the slowness of change in agriculture to embrace emergent technologies had actually caused it to lag behind. They argued that “our industry has probably been left behind in terms of tech compared to other industries, but farmers are slowly getting there” (Blog 2).

#### Ease of use

Secondly, in analysing the adjectives farmer interviewees used to describe newer, higher tech products versus and older, lower-tech equipment, there was a difference in perceived complexity. One of the farmer reflective blogs chose to frame its discussion around a series of different farm tasks, giving ‘high-tech’ or ‘low-tech’ ways of achieving it. He wrote, for example, about how a farmer could know that the grass in a field was optimal for a cow. “Low-tech’, he said “is letting cows into a field of grass that looks about right, high tech is going out with the plate meter” (Blog 1). On the process of getting cows in for milking, he wrote “low tech is a piece of string [on a gate], high tech is a timed gate that releases automatically to let the milkers head for the parlour” (Blog 1). For this farmer, therefore, there is a certain simplicity about the low-tech, as opposed to the complexity and sophistication of the high-tech. Other farmers in the interviews agreed. Whilst not all farmers felt that high-tech was challenging, phrases such as “complicated to use” or “more expensive” were used to refer to it, whereas low-tech was “simple, less expensive, durable”.

#### Sentimentality

There was also a discussion about sentimentally and the enjoyment of using or modifying older technology which had become so meaningful to farmers through everyday use over time. The 1994 tractor was referred to as “my father’s favourite” (F5) by one farmer who expressed their reluctance to replace it partially because of that.

#### Ease of repair

As well as performance and sentimentally, the ease of fixing or tinkering with older equipment was raised. Two farmers thought that older technologies “are easier to fix than electronics” (F1) or were more “easily fixable” (F3) than new ones. This is a theme that the literature has recently raised with reference to the ‘right to repair’ movement in agriculture (and other sectors) (Lumbard et al. 2020), as newer technologies become more complex to repair or require the company to carry out the repair under the purchasing agreement.

As well as the value of the old, there were perceived problems with the new. As documented in a large literature on farmer adoption of technology, there are a number of barriers that prevent the scaling of any new technology, including robots, drones, sensors, and other items associated with agriculture 4.0. These include (quotes mainly from interviews):

1. *Lack of skills to use new digital tools:* “I’m just probably not au fait with the software” (F5).
2. *Older age of farmers and lack of trust:* “there’s the stigma attached to technology when it’s very new, it’s hard to trust something that’s new that goes against the grain of the practice of farming that’s been carried out for numerous years” (Broker 3).
3. *Lack of performance and relevance of new tools:* “There are plenty of technologies that sound fab and definitely scratch the nerd nerves! But if they don’t work or if they aren’t picked up or they aren’t useful then they’re a waste of time. However cool they are” (Broker 10)
4. *Lack of interoperability between new and existing tools:* “Farmers do complain that some software systems, farm management systems, precision farming systems are hugely data hungry and they end up entering data of fields and yields and so forth and from different platforms, from their phone, from their iPhone, from their android phone, from a John Deere tractor, from a Claas tractor, from a Massey Ferguson combine and it all needs converting in some way to end up in the same spot” (Broker 6).
5. *High cost of some new tools:* “I can’t see that it’s going to go an awful lot further. The machine we currently have managed well for a decade without any further input, it’s still quite young and done very little work…and I really don’t see any point in spending 4 or 5 k to add something that will do the same job as it does now” (F4).
6. *Lack of rural connectivity:* the “mobile phone is probably the single more important too’, that is only ‘if a signal is available” (Farmer blog 3).
7. *Lack of enabling regulation:* “The specific example there is spot spraying with drones. So in China one of the most rapidly growing precision agriculture technologies is spot spraying with drones. I don’t know of any use of that technology in the UK. [redacted] has been trying to get a permission to do it for research purposes for years and hasn’t been able to do it even for research [because of regulation].” (Broker 8)

Our exploration of the everyday challenges faced by farmers in adopting new technologies adds to other factors in the literature (see review by da Silveria et al. 2021; also Barnes et al. 2019), thereby complicating the notion that agriculture 4.0 will be fast-paced and revolutionary. The pace of change depends on a number of factors that vary according to how technologies are domesticated, which is influenced by the adaptive capacity of individual farmers, as well as by farmers’ worldview (or imaginary) towards farming and innovation. For many reasons, some of which highlighted above, many farmers have no choice but to make use of the old and to re-purpose old or second-hand technologies, giving them a new lease of life in the absence of being able to use newer technologies.

### The seduction of the new

Much research in Science and Technology Studies has shown the beguiling nature of the new (e.g. Nordmann 2014). Emergent silver bullets, and the hyped discourses surrounding them, seem to offer a seductive technical solution to deeply entrenched social and political dilemmas (Rose et al. 2021b; Lajoie-O’Malley et al. 2020). As Duncan et al. (2021) argue in the context of agricultural futures, hyped narratives around the new have a material effect on which types of future are supported by policy instruments, such as research funding, grants for farmers, and incentives for the private sector. This makes one type of high-tech future more likely to pervade as compared to other futures which themselves may create a different set of winners and losers (Fleming et al. 2021; Ehlers et al. 2022). In our research, we asked farmers and innovation brokers to imagine the future of technology use on their farm (or on the farms they supported) as a means to further articulate a range of possible futures. What is clear from the evidence below is that many different technology trajectories are predicted and also that implicit or explicit normative choices to back certain visions without adequate inclusion risks the pursuit of unresponsible innovation.

**Fig. 9:**
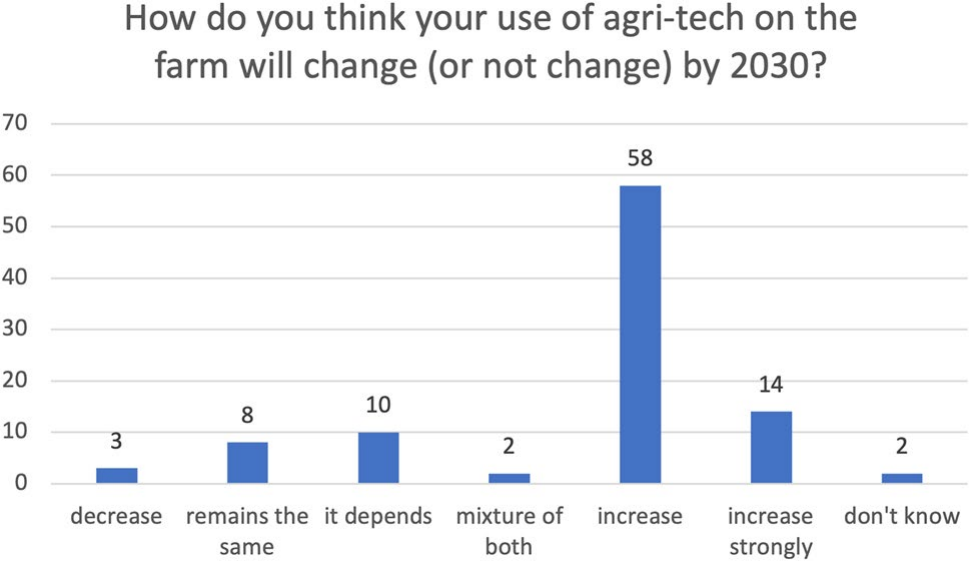
Graph showing farmer survey responses to the question ‘how will your use of agricultural technology change on your farm by 2030?’ (n-97, one farmer, response not clear)

In the survey, farmers were asked to predict how their use of technology would change by 2030. Coding answers into direction-based themes, Fig. 9 illustrates that most farmers thought that their use of technology (type not specified) would increase by 2030.

**Fig. 10:**
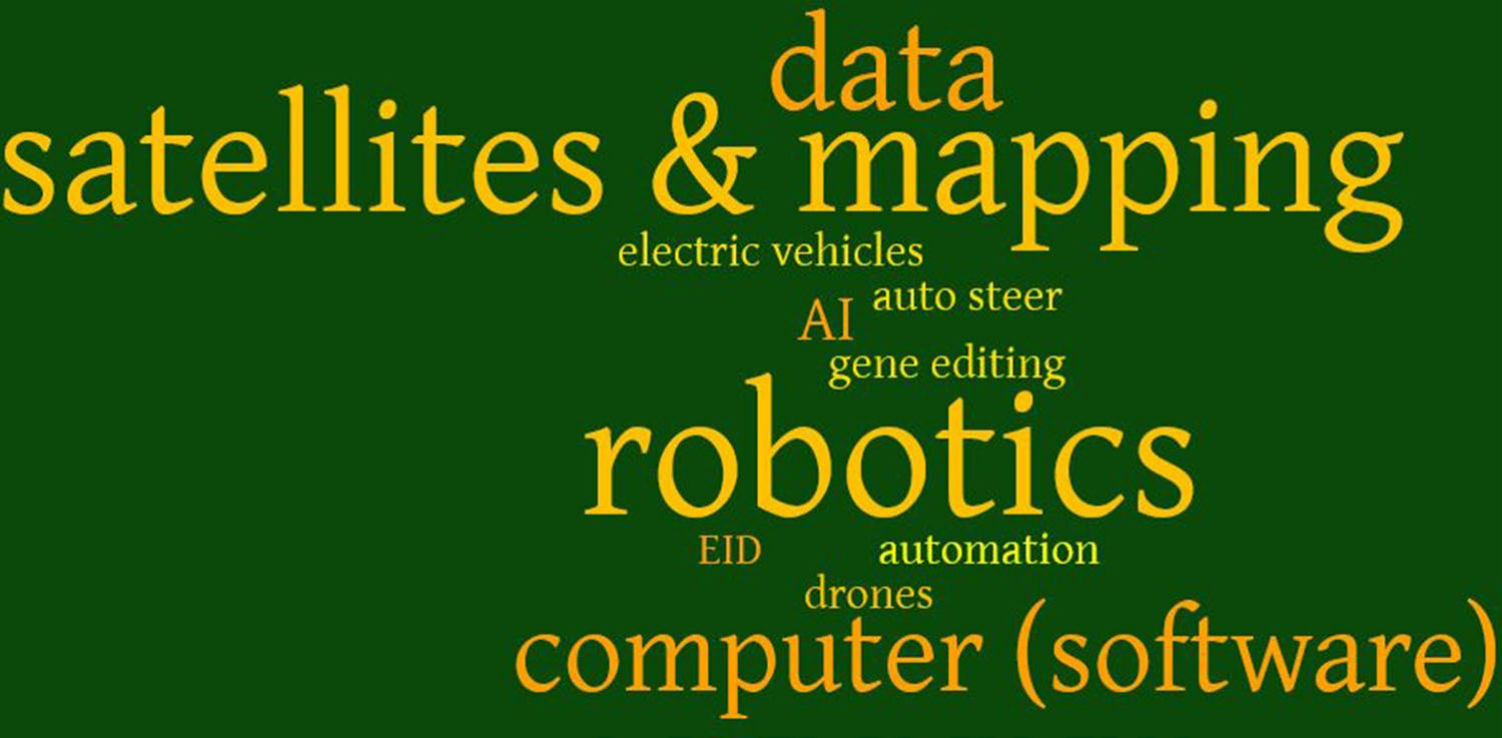
Technologies mentioned in the farmer survey if farmers gave answers within the codes ‘increase’ or ‘increase strongly’ in their responses

For those farmers who either said increase or strongly increase, they mentioned various technologies in the same comment. These are shown in Fig. 10. One farmer commented in the survey that they hoped “everything would be automated so I can stay in the house”, whereas another said in the interview that “the idea of looking out for a swarm of robots to me is an absolute horror” (F4). Several farmers predicted a divergence between high-cost and low-cost systems.

There were eleven farmers who felt their use of technology would decrease or remain the same by 2030, with a further ten saying it would depend on adoption factors discussed previously.

Innovation brokers were asked to predict the future of technology on the farm by 2030. The majority of respondents predicted much higher-tech visions, defined as the greater use of new technologies that are emerging at present. Several different visions were articulated:

- *Lower-input systems:* interviewees universally agreed that farms of the future would be lower input businesses and cited several technologies as playing a key role from gene editing, to robotics, and artificial intelligence.
- *More controlled environment agriculture:* several interviewees thought that we would produce more food away from existing farmland and therefore food-producing technologies would take food generation away from the traditional farm. One said: “do what the Dutch are doing on a bigger scale as we have more land. I know it horrifies some farm suppliers, but I think it's going to come along the way” (Broker 7).
- *High-tech futures, less small farms:* higher-tech farming futures were envisioned by several interviewees. Most spoke of the increasing use of robotics, drones, AI, and genetics. But, one interviewee thought that higher-tech visions would not come quickly:
  You can think too far sometimes in which you see things being done completely autonomously. Now robotics are far, far away from becoming fully autonomous and doing everything in practice because the landscape in farming, the variables in farming are changing all the time (Broker 3).

An innovation broker argued that change would be incremental:

It’s that idea around what’s a farmer and I think if you’re looking at the current zeitgeist of farming as we know it then it will no doubt be kind of incremental inventions that allow the status quo to be sustained for as long as possible. So there’s an interesting dichotomy there between, where the future is going and perhaps what farmers feel they need. (Broker 5)

Like farmers, the clear message is that several future visions of agriculture are likely to run concurrently, but the comment by broker 5 that “there’s an interesting dichotomy there between, where the future is going and perhaps what farmers feel they need” warrants further attention. Another broker argued the following:

I think we’re at a crossroads actually where funding is very focused on precision technologies and futuristic farming, which is at the expense of providing a portion of that funding for systems-based work. Funding is almost geared up at the moment to support smaller numbers of bigger, highly technological, high cash flow farms, whereas at the moment our system is very much a huge swath of smaller farms. (Broker 10)

Herein, we can see further empirical corroboration of the concerns laid out by Duncan et al. (2021); that a focus on supporting a particular vision of future agriculture – high-tech, high cash flow farms – may clash with what some “farmers feel they need”.

## Discussion

Our study has used the lens of the everyday to look beneath popular academic, policy and media discourses of a so-called agriculture 4.0 powered by emergent agricultural technologies (Barrett and Rose 2022; Duncan et al. 2021). In a bid to bring forth counter-narratives inspired by historical and sociological research on agricultural change, we tried to look past the visible and headline-grabbing markers of change (McWilliams 1941; Glover et al. 2019), instead focusing on what agricultural technology means to farmers and how they are experiencing and influencing the pace and directionality of change. We acknowledge that this is a relatively small study given aforementioned constraints, but we hope that it inspires further research to compile everyday encounters between farmers and technology to produce counter-narratives in an assumed period of revolutionary change. Three prominent themes emerge.

First, the pace and directionality of technological change on different farms across the world is unlikely to match the techno-optimism of agriculture 4.0 narratives. Our research adds further support to a non-linear conceptualisation of technological change on-farm (Baur and Iles 2022; Van der Veen 2010; Zagata et al. 2020). Whilst new technologies do replace the old, old technologies can replace the new, and retro-innovation or micro-invention can reinvigorate older items. This again depends on the personal circumstances of the farmer, how easy it is for them to adopt new technologies, or re-purpose old ones, and whether they want to stop using an item that still works or holds sentimental value. It could also be determined by the type of production system they wish to be a part of. Further research is required to shed light on how agriculture 4.0 progresses differently across and within different sectors and regions.

Secondly, whilst there is a large breadth of literature on barriers to adoption of technology by farmers (see review by Silveira et al. 2021), there is relatively little on the value of old versus new tools. Our work illustrates the value of the old for reasons of performance, ease of use, sentimentality, and ease of repair, alongside the unattainability of the new to some farmers and this explains the existence of counter-narratives to agriculture 4.0. The differing adaptive capacity of individual farmers for many reasons, including variations in skills, investment ability, connectivity, age, and sector mean that some farmers are better able to take advantages of agriculture 4.0 technologies than others (Asfaw et al. 2016; Eakin et al. 2016; Lowitt et al. 2015). If we take future adoption of autonomous robotics as an example, farmers with better 4G and 5G connectivity, existing suitable building and other infrastructure that could be adapted to storing and charging robots, and less restrictive regulation which could diminish value of labour substitution (e.g. laws requiring human supervision of robots, Lowenberg-deBoer et al. 2021), are most likely to adopt these new tools. This leads us onto our final theme elucidating the winners and losers created by supporting one future vision of agriculture over another.

Finally, as noted by farmers and innovation brokers, there are many different possible agricultural futures and perceptions about the desirability of those futures. Through the use of different policy instruments, favouring a dominant narrative of agriculture 4.0 higher-tech excludes, or makes less likely, other visions of the future, which themselves create a set of winners and losers. This makes it easier for some realities to persist than others (Duncan et al. 2021). If we are to pursue responsible agricultural futures, as has been argued in the literature (e.g. Bronson 2019; Eastwood et al. 2019), the governance of sustainable transitions needs to foster the substantive inclusion of stakeholder communities (Bronson 2019; Rose and Chilvers 2018). Crucial to this process is the inclusion of everyday encounters between farmers and technology, bringing forth less powerful voices. Further research exploring these everyday encounters in different places, and on farms of varying sizes and types of enterprise, is an important step in documenting how farmers are experiencing technological change and in guiding the responsible use of policy instruments to support different farming communities. These less powerful, or ‘harder to reach’ (Hurley et al. 2022), voices tend to be more challenging for policy-makers to include in traditional processes of policy engagement (de Boon et al. 2022), and thus researchers and skilled intermediaries have a role to play in highlighting perspectives that may otherwise be missed. These perspectives could lead to the use of policy instruments that help less powerful farmers to adapt better to changing conditions, level the playing field of innovation to boost smaller- and medium sized enterprise competitivity (Birner et al. 2021), or to encourage developers of technology to design technology with smaller-scale farmers in mind, rather than developing tacitly or deliberately with larger farmers in mind (see ‘Diversity by Design’ project^9^).

## Concluding remarks: reflecting on everyday tech encounters

In using the everyday to compile grounded experiences of technological change, we show that academic, policy, and media narratives need to go beyond grand, pervasive stories of technology revolutions. We have illustrated the need to capture the local, everyday enactment of technology on farms and consider the microhistories of change, rather than arbitrarily demarcating headline moments of progress. As we have argued in this paper, the inclusion of grounded everyday encounters (Glover et al. 2019) to show how farmers ‘live with’ technology (Lundström and Lindblom 2021) reveals the non-linearity of change. This challenges not only the myth of agriculture 4.0, but also associated policy and funding support that may fail to account for the different paces and directionality at which different farmers experience change. There would be a certain amount of delusion in only supporting the development and the adoption of the new (Vinsel and Russell 2020).

The everyday encounters that affect domestication cannot be studied without a close engagement with the realities of how farmers experience technology on the farm. Our mixed methods approach of interviewing, surveys, social media, and photography could be joined by others that are suited to compiling microhistories of everyday encounters between technology and farmers. Chief amongst these would be the greater use of ethnography, but also archival research to investigate further how change has been experienced in the past. Whilst agricultural historians and sociologists have laid the groundwork for critiques of agricultural revolutions, the idea of swift periods of technological change, of the early adopter, persist. This is partially, of course, because the myth of rapid change perpetuated by revolutionary technologies is seductive to those who gain most from specific trajectories. But, it could also point to the ineffectiveness of scholarly attempts to conduct and disseminate the research needed to debunk the myth and create convincing and evidence-based counter-narratives. We argue that greater multi-disciplinarity, centred around observations of the everyday, would strengthen our understanding of how and why technological change occurs on farm, which in turn may lead to wider dissemination of the counter-narratives required to shape responsible agricultural futures.

## Data Availability

Interview and survey data are not available. This was a small project with no funding available for data anonymisation and archiving. As such, respondents were not asked to give consent for data archiving. Innovation broker interviews would not have been archived even with resources due to the relatively small sample population and ability to identify respondents.

## Footnotes

1 Defined as a ‘collectively imagined form[] of social life and social order reflected in the design and fulfillment of nation-specific scientific and/or technological projects’ (Jasanoff and Kim 2009, p. 120).

2 https://farmhack.org/tools.

3 How quickly technological change occurs and whether always in a linear fashion, old to new.

4 https://farmhack.org/tools.

5 https://farmhack.org/tools/home-built-no-till-seed-drill.

6 https://farmhack.org/tools/water-wheel-transplanter.

7 https://farmhack.org/tools/livestock-weigh-scales.

8 https://www.the4ar.com/.

9 https://scienceandsocietycollective.com/diversity-by-design-emergent-agricultural-technologies-for-small-scale-farming/.

## References

[CR1] Asfaw S, McCarthy N, Lipper L, Arslan A, Cattaneo A (2016). What determines farmers’ adaptive capacity?. Empirical Evidence from Malawi. Food Security.

[CR2] Barnes AP, Soto I, Eory V, Beck B, Balafoutis A (2019). Exploring the adoption of precision agricultural technologies: A cross regional study of EU farmers. Land Use Policy.

[CR3] Barrett H, Rose DC (2022). Perceptions of the Fourth Agricultural Revolution: What’s In, What’s Out, and What Consequences are Anticipated?. Sociologia Ruralis.

[CR4] Basu S, Omotubora A, Beeson M, Fox C (2020). Legal framework for small autonomous agricultural robots. AI & Society.

[CR5] Baur P, Iles A (2022). Replacing humans with machines: A historical look at technology politics in California agriculture. Agriculture and Human Values.

[CR6] Birner R, Daum T, Pray C (2021). Who drives the digital revolution in agriculture? A review of supply-side trends, players and challenges. Applied Economics Perspectives and Policy.

[CR7] Borup M, Brown N, Konrad K, van Lente J (2006). The sociology of expectations in science and technology. Technology Analysis and Strategic Managementm.

[CR8] Bronson K (2019). Looking through a responsible innovation lens at uneven engagements with digital farming. NJAS—Wageningen Journal of Life Sciences.

[CR9] Brooks S (2021). Configuring the digital farmer: A nudge world in the making?. Economy and Society.

[CR10] Bryman A (2008). Social research methods.

[CR11] Burchardt J (2007). Agricultural History, Rural History or Countryside History?. The Historical Journal.

[CR12] Cofré- Bravo G, Klerkx L, Engler A (2019). Combinations of bonding, bridging, and linking social capital for farm innovation: How farmers configure different support networks. Journal of Rural Studies.

[CR13] Da Silveira F, Lermen FH, Amaral FG (2021). An overview of agriculture 4.0 development: Systematic review of descriptions, technologies, barriers, advantages, and disadvantages. Computers and Electronics in Agriculture.

[CR14] Daum T (2021). Farm robots: Ecological utopia or dystopia?. Trends in Ecology and Evolution.

[CR15] Dauvergne P (2020). AI in the wild: Sustainability in the age of artificial intelligence.

[CR16] de Boon A, Sandström C, Rose DC (2022). Perceived legitimacy of agricultural transitions and implications for governance. Lessons learned from England’s post-Brexit agricultural transition. Journal of Rural Studies.

[CR17] De Laet M, Mol A (2000). The Zimbabwe Bush Pump: Mechanics of a Fluid Technology. Social Studies of Science.

[CR18] de Oca Munguia OM, Pannell DJ, Llewellyn R, Stahlmann-Brown P (2021). Adoption pathway analysis: Representing the dynamics and diversity of adoption for agricultural practices. Agricultural Systems.

[CR19] Duncan E, Glaros A, Ross DZ, Nost E (2021). New but for whom? Discourses of innovation in precision agriculture. Agriculture and Human Values.

[CR20] Duncan E, Rotz S, Magnan A, Bronson K (2022). Discipling land through data: The role of agricultural technologies in farmland assetization. Sociologia Ruralis.

[CR21] Eakin H, York A, Aggarwal R, Waters S, Welch J, Rubiños C, Smith-Heisters S, Bausch C, Anderies JM (2016). Cognitive and institutional influences on farmers’ adaptive capacity: Insights into barriers and opportunities for transformative change in central Arizona. Regional Environmental Change.

[CR22] Eastwood C, Klerkx L, Ayre M, Dela Rue B (2019). Managing Socio-Ethical Challenges in the Development of Smart Farming: From a Fragmented to a Comprehensive Approach for Responsible Research and Innovation. Journal of Agricultural and Environmental Ethics.

[CR23] Edgerton D (1999). From innovation to Use: Ten Eclectic Theses on the Historiography of Technology. History and Technology: An International Journal.

[CR24] Edgerton D (2006). The shock of the old: Technology and global history since 1900.

[CR25] Ehlers M-H, Finger R, El Benni N, Gocht A, Sørensen CAG (2022). Scenarios for European agricultural policy-making in the era of digitalisation. Agricultural Systems..

[CR26] Fielke S, Taylor B, Jakku E (2020). Digitalisation of agricultural knowledge and advice networks: A state-of-the-art review. Agricultural Systems.

[CR27] Fleming A, Jakku E, Fielke S, Taylor BM, Lacey J (2021). Foresighting Australian digital agricultural futures: Applying responsible innovation thinking to anticipate research and development impact under different scenarios. Agricultural Systems.

[CR28] Gardezi M, Adereti DT, Stock R, Ogunyiola A (2022). In pursuit of responsible innovation for precision agriculture technologies. Journal of Responsible Innovation.

[CR29] Glover D, Sumberg J, Ton G, Andersson J, Badstue L (2019). Rethinking technological change in smallholder agriculture. Outlook on Agriculture.

[CR30] Gosden C, Marshall Y (1999). The cultural biography of objects. World Archaeology.

[CR31] Hurley P, Lyon J, Hall J, Little R, Tsouvalis J, White V, Rose DC (2022). Co-designing the environmental land management scheme in England: The why, who and how of engaging ‘harder to reach’ stakeholders. People and Nature.

[CR32] Ingram J (2008). Agronomist-farmer knowledge encounters: An analysis of knowledge exchange in the context of best management practices in England. Agriculture and Human Values.

[CR33] Jasanoff S, Kim SH (2009). Containing the atom: Sociotechnical imaginaries and nuclear power in the United States and South Korea. Minerva.

[CR34] Kerridge E (1969). The Agricultural Revolution Reconsidered. Agricultural History.

[CR35] Klerkx L, Jakku E, Labarthe P (2019). A review of social science on digital agriculture, smart farming and T agriculture 4.0: New contributions and a future research agenda. NJAS—Wageningen Journal of Life Sciences.

[CR36] Klerkx L, Rose D (2020). Dealing with the game-changing technologies of Agriculture 4.0: How do we manage diversity and responsibility in food system transition pathways?. Global Food Security.

[CR37] Kumar R, Lorek T, Olsson TC, Sackley N, Schmalzer S, Soto G (2017). Roundtable: New Narratives of the Green Revolution. Agricultural History.

[CR38] Lajoie-O’Malley A, Bronson K, van der Burg S, Klerkx L (2020). The future(s) of digital agriculture and sustainable food systems: An analysis of high-level policy documents. Ecosystem Services.

[CR40] Lowenberg-DeBoer J, Erickson B (2019). Setting the Record Straight on Precision Agriculture Adoption. Agronomy Journal.

[CR41] Lowenberg-DeBoer L, Behrendt K, Ehlers M-H, Dillon C, Gabriel A (2021). Lessons to be learned in adoption of autonomous equipment for field crops. Applied Economics Perspectices and Policy.

[CR42] Lowitt K, Hickey GM, Saint Ville A, Raeburn K, Thompson-Colón T, Laszlo S, Phillip LE (2015). Factors affecting the innovation potential of smallholder farmers in the Caribbean Community. Regional Environmental Change.

[CR43] Lumbard, K., V. Ahuja, and M. Snell. 2020. Open agriculture and the right-to-repair community movement. In *MWAIS 2020 Proceedings*, vol. 13.

[CR44] Lundström C, Lindblom J (2018). Considering farmers’ situated knowledge of using agricultural decision support systems (AgriDSS) to Foster farming practices: The case of CropSAT. Agricultural Systems.

[CR45] Lundström C, Lindblom J (2021). Care in dairy farming with automatic milking systems, identified using an Activity Theory lens. Journal of Rural Studies.

[CR46] Marres N (2015). Material participation: Technology, the environment and everyday publics.

[CR47] McCampbell M, Adewopo J, Klerkx L, Leeuwis C (2021). Are farmers ready to use phone-based digital tools for agronomic advice? Ex-ante user readiness assessment using the case of Rwandan banana farmers. The Journal of Agricultural Education and Extension.

[CR48] McWilliams C (1941). Farms into factories: Our agricultural revolution. The Antioch Review.

[CR49] Mehrabi Z, McDowell MJ, Ricciardi V, Levers C, Martinez JD (2021). The global divide in data-driven farming. Nature Sustainability.

[CR50] Miles C (2019). The combine will tell the truth: On precision agriculture and algorithmic rationality. Big Data & Society.

[CR51] Nordmann A (2014). Responsible innovation, the art and craft of anticipation. Journal of Responsible Innovation.

[CR52] Nutch F (1996). Gadgets, Gizmos, and Instruments: Science for the Tinkering. Science, Technology, & Human Values.

[CR53] Nyborg S (2015). Pilot Users and Their Families: Inventing Flexible Practices in the Smart Grid. Science & Technology Studies.

[CR54] Nye DE (1994). American technological sublime.

[CR55] Oldrup HH, Carstennsen TA (2021). Producing geographical knowledge through visual methods. Geografiska Annaler: Series b, Human Geography.

[CR56] Pielke R, Linnér B-A (2019). From Green Revolution to Green Evolution: A critique of the political myth of averted famine. Minerva.

[CR57] Prause L (2021). Digital agriculture and labor: A few challenges for social sustainability. Sustainability.

[CR58] Rijswijk K, Klerkx L, Turner JA (2019). Digitalisation in the New Zealand Agricultural Knowledge and Innovation System: Initial understandings and emerging organisational responses to digital agriculture. NJAS—Wageningen Journal of Life Sciences.

[CR60] Rose DC, Chilvers J (2018). Agriculture 4.0: Broadening responsible innovation in an era of smart farming. Frontiers in Sustainable Food Systems.

[CR61] Rose DC, Lyon J, de Boon A, Hanheide M, Pearson S (2021). Responsible development of autonomous robotics in agriculture. Nature Food.

[CR62] Rose DC, Morris C, Lobley M, Winter M, Sutherland WJ, Dicks LV (2018). Exploring the spatialities of technological and user re-scripting: The case of decision support tools in UK agriculture. Geoforum.

[CR63] Rose DC, Sutherland WJ, Parker C, Lobley M, Winter M, Morris C, Twining S, Ffoulkes C, Amano T, Dicks LV (2016). Decision support tools for agriculture: Towards effective design and delivery. Agricultural Systems.

[CR64] Rose DC, Wheeler R, Winter M, Lobley M, Chivers C-A (2021). Agriculture 4.0: Making it work for people, production, and the planet. Land Use Policy.

[CR65] Rotz S, Gravely E, Mosby I, Duncan E, Finnis E, Horgan M (2019). Automated pastures and the digital divide: How agricultural technologies are shaping labour and rural communities. Journal of Rural Studies.

[CR66] Schaffer S (2011). Easily cracked: Scientific instruments in states of disrepair. Isis.

[CR67] Schillings J, Bennett R, Rose DC (2021). Animal welfare and other ethical implications of Precision Livestock Farming Technologies. CABI Agriculture and Bioscience.

[CR68] Shiva V (2016). The violence of the Green Revolution: Third world agriculture, ecology, and politics.

[CR69] Smith HE, Sallu SM, Whitfield S, Gaworek-Michalczenia MF, Recha JW (2021). Innovation systems and affordances in climate smart agriculture. Journal of Rural Studies.

[CR70] Streed, E., B. Tomlinson, M. Kantar, and B. Raghavan. 2021. How Sustainable is the Smart Farm? *LIMITS*. https://computingwithinlimits.org/2021/papers/limits21-streed.pdf.

[CR71] Van der Veen M (2010). Agricultural innovation: Invention and adoption or change and adaptation?. World Archaeology.

[CR72] Vik J, Straete EP, Hansen BG, Naerland T (2019). The political robot – The structural consequences of automated milking systems (AMS) in Norway. NJAS – Wageningen Journal of Life Sciences.

[CR73] Vinsel, L., and A. Russell. 2020. The innovation deulsion: How our obsession with the new has disrupted the work that matters most. *Currency*. New York: Penguin Random House.

[CR74] Wiseman L, Sanderson J, Zhang A, Jakku E (2019). Farmers and their data: An examination of farmers’ reluctance to share their data through the lens of the laws impacting smart farming. NJAS Wageningen Journal of Life Sciences.

[CR75] Zagata L, Sutherland L-A, Hrabák J, Lostak M (2020). Mobilising the past: Towards a conceptualisation of retro-innovation. Sociologia Ruralis.

